# New Technologies to Study Functional Genomics of Age-Related Macular Degeneration

**DOI:** 10.3389/fcell.2020.604220

**Published:** 2021-01-11

**Authors:** Tu Nguyen, Daniel Urrutia-Cabrera, Roxanne Hsiang-Chi Liou, Chi D. Luu, Robyn Guymer, Raymond Ching-Bong Wong

**Affiliations:** ^1^Centre for Eye Research Australia, Royal Victorian Eye and Ear Hospital, East Melbourne, VIC, Australia; ^2^Ophthalmology, Department of Surgery, University of Melbourne, Melbourne, VIC, Australia

**Keywords:** age-related macular degeneration, retina, CRISPR/Cas, induced pluripotent stem cell models, single cell transcriptomics, organoids

## Abstract

Age-related macular degeneration (AMD) is the most common cause of irreversible vision loss in people over 50 years old in developed countries. Currently, we still lack a comprehensive understanding of the genetic factors contributing to AMD, which is critical to identify effective therapeutic targets to improve treatment outcomes for AMD patients. Here we discuss the latest technologies that can facilitate the identification and functional study of putative genes in AMD pathology. We review improved genomic methods to identify novel AMD genes, advances in single cell transcriptomics to profile gene expression in specific retinal cell types, and summarize recent development of *in vitro* models for studying AMD using induced pluripotent stem cells, organoids and biomaterials, as well as new molecular technologies using CRISPR/Cas that could facilitate functional studies of AMD-associated genes.

## Introduction

Age-related macular degeneration (AMD) is the leading cause of central vision loss among people over 50 years old in developed countries (Klein et al., 1992; Mitchell et al., 1995). Due to the increase of the aging population, the prevalence of AMD is estimated to affect 196 million globally in 2020, reaching 288 million by 2040 (Wong et al., 2014). Early and intermediate AMD are characterized by minimal visual symptoms, pigmentary changes in the macula and formation of drusen beneath the retina (Hernández-Zimbrón et al., 2018). Advanced AMD is classified into two types: atrophic and neovascular. In atrophic AMD, gradual vision loss occurs due to retinal pigment epithelium (RPE) degeneration and photoreceptor death. Neovascular AMD often causes sudden vision loss due to the formation of abnormal, new blood vessels, usually developing in the choroid and invading through Bruch’s membrane into the neuroretina. When this occurs there is usually fluid leakage, hemorrhage, and if left untreated, irreversible scarring will occur which affects the RPE and photoreceptors at the macula, ultimately leading to severe loss of central vision (Velez-Montoya et al., 2014). Research on AMD pathogenesis has been largely focused on the contribution of RPE and Bruch’s membrane, but lately there is also renewed interest in the role of the choroid. There is increasing evidence suggesting that pathological changes in the choroid may play an early role in the pathogenesis of AMD (Farazdaghi and Ebrahimi, 2019). For example, loss of choriocapillaris endothelial cells is one of the earliest detectable events in this disease, which can drive progression to more advanced stages due to subsequent loss of metabolic support to the outer retina (Chirco et al., 2017). Altogether, photoreceptor cells, RPE, Bruch’s membrane and choroid depend upon each other to maintain normal vision. Future research should explore all these domains to gain a better insight into factors at play throughout the course of the disease.

**FIGURE 1:**
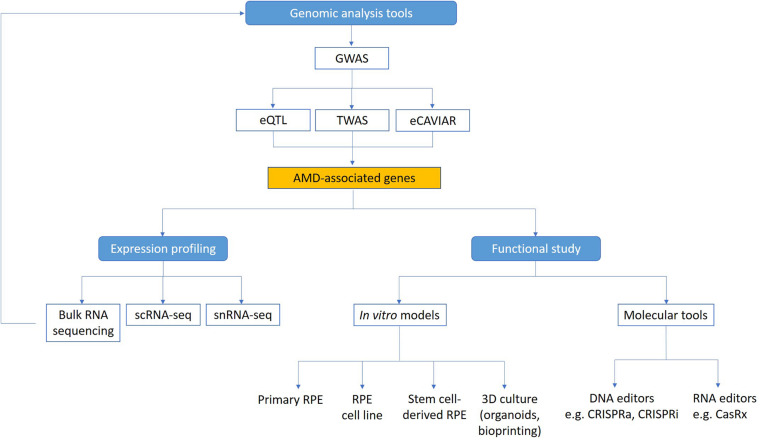
Technologies to study functional genomics of AMD. Genome-wide analysis methods (GWAS, eQTL, TWAS, and eCAVIAR) can be used to identify candidate genes linked to AMD. Expression of these genes in the retina can be mapped using transcriptomic technologies. Functional study of AMD-associated genes can be facilitated using *in vitro* AMD models and molecular tools. scRNA-seq, single cell RNA sequencing; snRNA-seq, single nuclei RNA sequencing.

Although many AMD associated genes have been identified from genome-wide association study (GWAS), currently there is a lack of understanding of the biological significance of many of these genes and their functions in AMD pathophysiology. This remains a major roadblock to finding specific treatments for AMD, especially in terms of a preventative therapy or cure. In neovascular AMD, past treatment strategies with laser and photodynamic therapy, as well as current therapies using anti-VEGF treatments are not treating the underlying cause, just the consequences of the disease (Colquitt et al., 2008; Brown et al., 2009; Guymer et al., 2014; Khanna et al., 2019). Thus, even when appropriate treatments are delivered, with time atrophy and fibrosis will often affect the outcomes and lead to longer term vision loss (Jaffe et al., 2019). Furthermore, there is no approved treatment available for atrophic AMD (Hernández-Zimbrón et al., 2018). This is partly because we are only beginning to uncover the complex interaction between genetics, environmental factors and the aging process that contribute to AMD. A deeper insight into how these different components play a role in pathogenesis will help us develop more effective treatments and preventative strategies for AMD. Critically, two key questions need to be addressed in order to translate the knowledge on AMD genetics and identify new therapeutic targets: 1) What are the expression profiles of AMD genes in the outer retina and choroid? and 2) What are the functions of these AMD-associated genes? Understanding in more detail the contribution of genes that underlie AMD pathophysiology could help us discover novel biomarkers to detect early AMD in those at risk of progression to vision-threatening late AMD, as well as identifying new therapeutic targets for AMD treatment. Here we summarize recent research on the genetic contributions to AMD, with a focus on improved genomic methods to identify novel AMD genes, as well as novel technologies including single-cell RNA sequencing (scRNA-seq), CRISPR, and *in vitro* stem cell models that will help us better understand how AMD-associated genes contribute to disease mechanisms (Figure 1).

## Genome-Wide Analysis to Study Genetic Contributions to AMD

Advances in genome-wide analyses have helped tremendously in our understanding of the genetics of AMD. Several genome-wide analysis techniques have been developed to identify genetic associations with diseases, such as GWAS, which detects single nuclear polymorphisms (SNPs) associated with diseases (Chang et al., 2018). Using GWAS, studies have now identified 69 SNPs related to AMD (Fritsche et al., 2013, 2016; Han et al., 2020), including loci that confer major susceptibility such as *CFH* and *ARMS2/HTRA1* (Hageman et al., 2005; Klein et al., 2005; Rivera et al., 2005).

*CFH* is an important inhibitor of the alternative complement pathway (Rivera et al., 2005). Within the *CFH* gene, the Y402H variant appears to have the strongest association to AMD susceptibility (Edwards et al., 2005; Hageman et al., 2005; Haines et al., 2005; Klein et al., 2005; Souied et al., 2005; Zareparsi et al., 2005; Magnusson et al., 2006). This polymorphism has been demonstrated to decrease CFH binding to C reactive protein, heparin, Streptococcus M protein, malondialdehyde epitopes, oxidized phospholipids, as well as heparan sulfate and dermatan sulfate glycosaminoglycans within Bruch’s membrane (Skerka et al., 2007; Yu et al., 2007; Ormsby et al., 2008; Clark et al., 2010; Weismann et al., 2011; Shaw et al., 2012; Molins et al., 2016). This reduced binding can cause inappropriate complement regulation, which results in chronic inflammation, abnormal physiological homeostasis and cell damage. The link between *CFH* and AMD suggests an inflammatory role in the pathogenesis of AMD, with increased complement cascade activity promoting AMD development in genetically predisposed individuals (Hageman et al., 2005; Despriet et al., 2006).

In addition to *CFH*, the *ARMS2/HTRA1* loci are identified as a major contributor to the risk of developing AMD. The mechanisms as to how *ARMS2* plays a role in the progression of AMD are not well understood. A previous study suggested that *ARMS2* interacts with extracellular matrix proteins, including fibulins and EMILIN-2, which help assemble and stabilize extracellular matrix structures of the Bruch’s membrane (Kortvely et al., 2010). *ARMS2* has also been shown to participate in the phagocytosis function of RPE, which may be a mechanism that contributes to the development of AMD (Xu et al., 2012). Another study has reported that *ARMS2* functions as a surface complement regulator – recombinant *ARMS2* binds to human apoptotic and necrotic cells and initiates complement activation to clear cellular debris (Micklisch et al., 2017). On the other hand, *HTRA1* encodes for a secreted protease that is involved in cell signaling, organization of extracellular matrix, and skeletal development (Hadfield et al., 2008; Mauney et al., 2010; Vierkotten et al., 2011; Graham et al., 2013; Supanji et al., 2013). Additionally, *HTRA1* was reported to play a role in the development of a variety of cancers (Baldi et al., 2002; Chien et al., 2004; Mullany et al., 2011), Alzheimer’s disease and osteoarthritis (Grau et al., 2005, 2006; Launay et al., 2008).

While the role of *CFH* in the complement pathway has been well studied, there is still lack of consensus as to which gene or genetic variant in the *ARMS2/HTRA1* loci is functionally relevant to AMD pathology (Kanda et al., 2007; Kortvely et al., 2010; Xu et al., 2012; Micklisch et al., 2017; Tosi et al., 2017). Since *ARMS2/HTRA1* region exhibits high linkage disequilibrium, it remains controversial as to which of the two genes is causally linked to AMD pathogenesis by statistical means (Fritsche et al., 2008; Kanda et al., 2010; Yang et al., 2010; Friedrich et al., 2011). Several papers supported that *HTRA1* variants confer AMD risk (Tosi et al., 2017; Lin et al., 2018; Orozco et al., 2020), while other studies suggested *ARMS2* variants confer AMD risk (Kanda et al., 2007; Grassmann et al., 2017). Despite the strong linkage equilibrium, *ARMS2/HTRA1* region still displays some level of recombination, producing rare recombinant haplotypes (Grassmann et al., 2017). These recombinant haplotypes can be used to dissect a disease-associated genomic region in a similar fashion to gene mapping in monogenic diseases. One study has analyzed rare recombinant haplotypes in 16,144 AMD cases and 17,832 controls from the International AMD Genomics Consortium (Fritsche et al., 2016). Using logistic regression analysis, the findings suggested that variants in or close to *ARMS2* but not *HTRA1* are responsible for disease susceptibility (Grassmann et al., 2017).

Other than identifying major AMD susceptibility genes, GWAS has also been used to study the commonalities and differences between two advanced AMD subtypes, atrophic and neovascular (Fritsche et al., 2016). While these two subtypes were found to share the majority of genetic risk, one variant was identified to be specific to one subtype – a variant near *MMP9* was exclusively associated with neovascular but not with atrophic AMD. This finding suggested that individuals that have high risk of developing neovascular AMD also have high risk of atrophic AMD. Future therapeutic strategies should therefore aim to target variants that confer risk for both neovascular and atrophic AMD.

## Improved Methods to Identify AMD-Associated Genes by Integration of Gene Expression Data

Genome-wide association study is a valuable tool to identify disease associated loci, however, there are several limitations to this method. Firstly, although GWAS findings reveal SNPs that are associated with the disease, these SNPs do not necessarily establish causal variants and genes. Linkage disequilibrium can conceal causal variants responsible for the association, such as in the case of *ARMS2/HTRA1*, making it difficult to pinpoint variants that have an effect on the trait from GWAS data alone (Wainberg et al., 2019). Secondly, as SNPs are often in non-coding regions and may be up to 2 Mbps away from the affected genes, it is challenging to pinpoint the genes they impact on the disease (Brodie et al., 2016; Gusev et al., 2016; Zhu et al., 2016; Cannon and Mohlke, 2018). Moreover, this method yields a large number of hits, which could make functional characterization challenging (Tam et al., 2019). Importantly, additional studies are required to confirm if these causal genes are driving disease association.

To address this, GWAS studies can be complemented with expression quantitative trait loci (eQTL) studies to prioritize causal variants and genes at GWAS loci. Using gene expression dataset, eQTL identifies genetic variants that influence gene expression levels. These eQTLs can act in *cis* (locally) or in *trans* (at a distance) to a gene and affect gene expression at the level of transcription or translation (Majewski and Pastinen, 2011). To date, studies on AMD genetics have mostly focused on investigating *cis*-eQTLs (Ratnapriya et al., 2019; Han et al., 2020; Orozco et al., 2020) so it would be interesting to perform *trans*-eQTL studies in the future.

Recently, several methods were described to improve the integration of GWAS and eQTL studies. For instance, transcriptome-wide association study (TWAS) could be used to identify genes that mediate effects of genetic variants on phenotype, thereby prioritizing candidate causal genes and tissues underlying GWAS loci. This method involves the use of an expression panel such as eQTL to train a predictive model of expression from genotype. This model is then used to predict gene expression for individuals in the GWAS cohort and identify association with the trait (Wainberg et al., 2019). AMD-associated genes discovered by TWAS would provide better understanding into the influence of gene regulation on phenotypic consequences in this disease. TWAS has been used to investigate the relevance of AMD pathology in tissues other than the retina. Even though AMD pathology is observed in the posterior pole of the eye, several studies have suggested a systematic expression profile of AMD associated genes throughout the body (Morohoshi et al., 2009; Camelo, 2014; Cougnard-Grégoire et al., 2014; Paun et al., 2015; Kiel et al., 2017). A TWAS study discovered 106 genes significantly associated with AMD variants in at least one of 27 tissues investigated, which suggests the expression of AMD-associated genes is not only limited to retinal tissue but also observed in other tissues throughout the whole body (Strunz et al., 2020). Based on these results, future studies on therapeutic strategies for AMD should consider the systemic expression profile of AMD-associated genes and processes underlying AMD pathology.

Similarly, eCAVIAR is another method that uses GWAS and eQTL studies to determine colocalization of causal variants (Hormozdiari et al., 2016). eCAVIAR measures the probability that the same variant is causal in both a GWAS and eQTL study. The underlying concept is that if the same variant underlying GWAS locus also influences gene expression, then the relevant gene and tissue could contribute to disease mechanism.

Leveraging methods such as eQTL, TWAS, and eCAVIAR can help reveal new biological mechanisms of AMD GWAS risk loci. Using eQTL in human retinal samples, an elegant study by Ratnapriya et al. (2019) identified potential target genes in six novel AMD GWAS loci, and three additional genes using TWAS. In addition to eQTL and TWAS, this study also used eCAVIAR to prioritize the most plausible target genes. Lead GWAS SNPs were connected to specific target genes at known AMD-associated loci, including *B3GLCT, BLOC1S1, SH2B3, PLA2G12A, PILRB*, *TMEM199*, and *POLDIP2*. A subsequent study used co-localization of GWAS and eQTL and found 15 candidate causal genes for AMD, which included genes highly expressed in RPE, such as *TRPM1* and *TSPAN10*, and previously identified genes such as *BLOC1S1* and *TMEM199* (Orozco et al., 2020). These two studies demonstrated that the combination of different techniques allow better identification of genes associated with AMD.

## Potential Biological Role of Novel AMD-Associated Genes

Notably many of the recently identified AMD-associated genes are not well studied in the retina. Understanding their biological roles within the retina would provide critical insights in AMD pathogenesis. Interestingly, several novel AMD-associated genes have a potential role in protein degradation, including autophagy and ubiquitin-mediated degradation. For instance, *POLDIP2* has been identified as a significant gene for AMD susceptibility in several publications (Ratnapriya et al., 2019; Han et al., 2020; Strunz et al., 2020). It has been implicated in other diseases, such as Alzheimer’s disease, in which it was found to be a novel regulator of Tau aggregation (Kim et al., 2015). Overexpression of *POLDIP2* caused impairments in autophagy, which led to increased Tau aggregate formations. Conversely, downregulation of *POLDIP2* reduced reactive oxygen species (ROS)-induced Tau aggregation. Since there is evidence that suggests defective autophagy and oxidative stress contribute to AMD pathophysiology (Jarrett and Boulton, 2012; Mitter et al., 2012; Golestaneh et al., 2017), it would be interesting to investigate the association of *POLDIP2* to oxidative damage and autophagy and determine how it contributes to AMD pathogenesis.

Similarly, *BLOC1S1* was identified as a potential causal gene from the RDH5-CD63 AMD risk locus in two eQTL studies (Ratnapriya et al., 2019; Orozco et al., 2020). BLOC1S1 is part of the octameric protein complex BLOC-1, which is associated with the biogenesis of organelles related to the endosome–lysosome system (Falcón-Pérez et al., 2002; Langemeyer and Ungermann, 2015). The BLOC-1 complex is associated with several biological processes, such as the sorting of synaptic vesicle proteins and postsynaptic receptors, cytoskeleton modulation, membrane fusion and macroautophagy, which malfunction could result in the development of a wide array of disorders (Hartwig et al., 2018). Notably, *BLOC1S1* was also identified as a potential causal gene using human RPE samples, where phagocytic (endocytic) and autophagy pathways play a major role in retinal homeostasis (Orozco et al., 2020). It is possible that disruption of these regulatory processes could result in accumulated stress for retinal cells within the macula, ultimately causing macular degeneration. Nevertheless, functional studies in retinal cells are required to validate the association of *BLOC1S1* in AMD pathogenesis.

*TMEM199* encodes an accessory component of the V-ATPase proton pump which is required for endo-lysosomal acidification. Human *TMEM199* mutations have been identified to disrupt Golgi homeostasis and cause glycosylation defects (Jansen et al., 2016). However, the role of *TMEM199* in the V-ATPase function remains unknown. In a recent study, Miles et al. reported that disruption of *TMEM199* resulted in intracellular iron depletion, thereafter impairing the activity of Iron(II) prolyl hydroxylase enzymes, which hydroxylate the HIF1α subunit and facilitate its proteasomal degradation. Prevention of HIF1α degradation leads to HIF activation, revealing an important role of *TMEM199* linking between the V-ATPase, iron metabolism and HIFs (Miles et al., 2017). As V-ATPase has been shown to engage in autophagic processes (Mijaljica et al., 2011), *TMEM199* may participate in response to chronic oxidative stress, hypoxia, and disturbed autophagy, which are crucial to the pathology of neovascular processes in AMD.

*NPLOC4* is another novel AMD gene that has been implicated in ubiquitin-mediated protein degradation. NPLOC4 forms a ternary complex with UFD1 and VCP, which binds ubiquitinated proteins and exports misfolded proteins from endoplasmic reticulum to the cytoplasm. As the NPLOC4-UFD1-VCP complex is involved in the proteasomal ubiquitin-dependent pathway, mutations of the complex have been reported to associate with multisystem proteinopathy that causes inclusion body myopathy, Paget’s disease of bone, and frontotemporal dementia (Blythe et al., 2019). In the retina, the ubiquitin-proteasome system and autophagy are essential for the degradation and recycling of cellular waste such as all-*trans* retinal in RPE cells (Blasiak et al., 2019). As such, disruption of *NPLOC4* may cause impaired protein degradation and contribute to the pathologic development of AMD. In summary, we have discussed several novel AMD-associated genes that could contribute to AMD pathogenesis through deregulation of protein degradation pathways. Biological roles of these genes in AMD as well as other novel genes can be explored using the technologies discussed in this review.

## New Transcriptomic Technologies to Map Expression of AMD-Associated Genes in the Retina

Recent advances in transcriptomic technologies have greatly facilitated our understanding of gene expression profiles of the human retina, providing a key step to better identify AMD-associated genes from GWAS and facilitate functional studies of these AMD-association genes. Previous studies have used bulk RNAseq to profile the human retina (Farkas et al., 2013; Whitmore et al., 2014; Pinelli et al., 2016; Hoshino et al., 2017). However, results of bulk RNA-seq represent an average signal of gene expression profile in all retinal cell types, as such gene expression in less abundant cell types would be obscured. Recent development of scRNA-seq technology overcomes this issue by resolving cell heterogeneity and profiling gene expression at a single cell level, providing a unique opportunity to reveal gene expression in specific retinal cell types. Using scRNA-seq, single cells from a complex tissue could be separated using microfluidic systems and tagged with a unique barcode to generate cDNA libraries for individual cells (Kulkarni et al., 2019). scRNA-seq technology can overcome the heterogeneity issue in complex tissue and provide a powerful method to analyze the transcriptome landscape of the retina.

Our team and others have recently utilized scRNA-seq to establish the transcriptome of major retinal cell types in the human retina (Lukowski et al., 2019; Menon et al., 2019; Voigt et al., 2019). Critically, these dataset could be used to study expression profiles of AMD-associated genes in the retina and characterize the effect of AMD on gene expression changes across different cell types and tissues. For instance, scRNA-seq has been used to analyze gene expression of 34 AMD risk loci identified by GWAS (Menon et al., 2019). Menon et al. (2019) found that within the retina, the majority of genes surrounding the 34 risk loci were expressed in Müller glia and astrocytes, including leading AMD genes (nearest gene to lead GWAS variant) such as *CFI*, *VEGFA*, *TIMP3*, and *COL4A3*. They also identified the cell types within the retina that are most predictive of AMD risk are cone photoreceptors, glial, and vascular cells. These results suggest that in addition to photoreceptors and RPE, which are the major cell types affected by AMD, glia and vascular cells are also potentially important in AMD pathogenesis.

On the other hand, single-nucleus RNA-seq (snRNA-seq) provides an alternative method for gene expression profiling in complex tissues from frozen samples at single cell levels (Grindberg et al., 2013). Compared to scRNAseq, snRNA-seq analyze gene expression within the nuclei instead of intact cells. It should be noted that there could be potential differences between the RNA type and expression levels between nucleus and cytosol. As observed in a previous study comparing nuclear and whole cell transcriptome in mouse neurons at single cell levels, a subset of genes associated with mitochondrial respiration was almost exclusively detected in the whole cell transcriptome (Lake et al., 2017). In addition, since nascent transcripts are naturally abundant in nuclei, there is a difference in maturity levels of transcripts detected between the nucleus and cytosol. Shorter genes were better detected in whole cells while longer genes showed better detection in the nuclei, and as a result, an additional normalization step was required to reduce technical bias. In regards to AMD, recent snRNA-seq studies have established the transcriptome of the macula and peripheral regions of the human retina (Liang et al., 2019; Orozco et al., 2020). In particular, Orozco et al. identified substantial gene expression differences between the macula and peripheral retina, including several AMD-associated genes such as *CFI*, *HTRA1*, *B3GLCT*, *TSPAN10*, and *PILRA* (Orozco et al., 2020). In addition, the authors also showed that the majority of AMD-associated genes are present in non-neuronal cells: 38% of the genes are expressed in RPE cells, 29% in Müller glia, and 27% in astrocytes. These studies highlighted the potential of using snRNA-seq to identify the retinal cells that contribute to AMD pathogenesis.

In summary, single cell transcriptomics provided useful tools in understanding the genetic signals within the retina and identifying the potential cell types within the retina that contribute to AMD pathogenesis. Future studies using single cell transcriptomics to analyze the retinal gene expression profiles in AMD patients could allow us to map the molecular deregulation in specific retinal cells that lead to AMD.

## New *in vitro* Models to Facilitate Functional Study of AMD-Associated Genes

Recent development of *in vitro* cell systems provides better models to study AMD. This includes the use of primary RPE and choroidal endothelial cells, immortalized cell lines, and cells derived from embryonic stem cells (ESCs) or induced pluripotent stem cells (iPSCs) (Malek et al., 2020). These *in vitro* models are useful for evaluating various cellular responses associated with AMD development, such as morphology, metabolism, cell proliferation, oxidative stress, and cell viability.

Primary RPE cells can be obtained from human donor eyes to study AMD, including human fetal RPE cells and adult RPE cells (Malek et al., 2020). However, there are several limitations associated with using primary cells. Firstly, access to donor human eyes can be limited and establishing primary culture is time-consuming and labor-intensive. Secondly, there could be potential variability between different donors. Finally, primary cells have limited proliferative capacity and will eventually senescence in culture. To address this, several human RPE cell lines have been developed to facilitate *in vitro* studies, which include spontaneously transformed cell lines H80HrPE-6, ARPE-19, D407 RPE, RPE-340, as well as immortalized cell lines hTERT RPE-1, h1RPE-7, and h1RPE-116 (Kuznetsova et al., 2014). In particular, ARPE-19 is the most commonly used human RPE cell line to date, which has morphological and functional characteristics of native RPE, including the abilities to phagocytose the photoreceptor outer segments (POS) (Finnemann et al., 1997) and secret endogenous growth factors (Ablonczy et al., 2011). However, ARPE-19 do not exhibit several properties of the native RPE, such as the absence of pigmentation and reduced expression levels of some RPE markers (e.g., *RPE65* and *CRALBP*) and low transepithelial resistance (Ablonczy et al., 2011). Treatment with nicotinamide could improve some RPE characteristics of ARPE-19, such as expression of RPE65 and better polarization (Hazim et al., 2019). Despite these limitations, ARPE-19 cell line represents a valuable *in vitro* model to study functions of AMD genes in RPE since it is commercially available and easy to culture.

Besides primary cells and cell lines, pluripotent stem cells provide an attractive *in vitro* model to study AMD. Somatic cells from patients can be reprogrammed into iPSC (McCaughey et al., 2016), which can be subsequently differentiated into the clinically relevant cell types for AMD, such as RPE and photoreceptors. iPSC provided a feasible strategy to derive patient-specific models to study ocular diseases, as we described previously (Khan et al., 2016; Hung et al., 2017; Wong et al., 2017). In particular, stem cell-derived RPE cells demonstrate similar characteristics to those of native RPE, such as polygonal morphology, pigmentation, phagocytosis of POS and ability to metabolize vitamin A (Malek et al., 2020). iPSC-derived RPE are also highly similar to human fetal RPE in terms of gene expression and enriched transcription factor motif profiles (Lidgerwood et al., 2016; Galloway et al., 2017; Smith et al., 2019). Also, an elegant study by Hallam et al. (2017) reported an iPSC model from AMD patients homozygous for low- and high-risk CFH (Y402H) polymorphism. Interestingly, iPSC-derived RPE cells derived from patients with high-risk genotypes resembled many key AMD features such as elevated inflammation and cellular stress, impaired autophagy, deposition of drusen-like deposits, and lipid droplets accumulation. Collectively, these studies support the feasibility of using patient-specific iPSC-derived RPE as an *in vitro* model to better study macular degeneration.

There are limitations to the use of monolayer RPE cultures to study AMD, as they cannot recapitulate the complex retina structure with supporting tissues around the RPE, such as photoreceptors, Bruch’s membrane and choroid. To address this, several three-dimensional (3D) culture systems are developed, providing better models to investigate cellular interaction within the microenvironment of the retina, such as retinal organoids derived from pluripotent stem cells. Early reports demonstrated that ESCs or iPSCs can self-organize and form retinal organoids, which are 3D structures that recapitulate *in vivo* development of the retina (Eiraku et al., 2011; Nakano et al., 2012). Subsequently, several groups have modified the retinal organoid differentiation process by promoting cell specification using exogenous transcription factors or modulators/growth factors of signaling pathways (Zhou et al., 2015; Hasegawa et al., 2016; Völkner et al., 2016). For example, inhibition of Notch signaling could promote retinal differentiation and generate retinal organoids that are enriched with photoreceptors (Völkner et al., 2016). Photoreceptors with mature features can also be derived using retinal organoids, including the connecting cilia, inner and outer segments (Gonzalez-Cordero et al., 2017). However, a key limitation of the organoid differentiation method is that it is time-consuming, with up to 6–7 months needed for the derivation of light sensitive photoreceptors (Zhong et al., 2014). Further research to understand the differentiation signaling could improve the kinetic of the retinal organoid method. Altogether, patient-specific organoid system provides a unique 3D model which can be used to study the influences of genetic signaling on cell–cell interaction between retinal cells.

The use of synthetic and natural polymeric scaffolds provided an alternative method to model the Bruch’s membrane in a 3D culture system (Trese et al., 2012). Previous studies showed that the Bruch’s membrane can be mimicked using porous scaffold constructed with polycaprolactone (McHugh et al., 2014; Xiang et al., 2014). This biomaterial creates a highly porous membrane which has been demonstrated to promote growth of RPE cells, as well as cell–cell interaction and migration. In particular, “artificial Bruch’s membrane” can be incorporated with human RPE cells and primate choroidal cells to construct a 3D co-culture model to better model the complexity of the retina (Shokoohmand et al., 2017). Similarly, cell sheet engineering can be applied to construct artificial substitutes of the choroid, by harvesting sheets of cultured cells along with their extracellular matrix, and integrating them to create tissue-like structures (Mokhtarinia et al., 2018). For instance, Djigo et al. (2019) have tissue-engineered a choroidal stromal model by stacking sheets of choroidal stroma fibroblasts and their extracellular matrix together. In addition, this choroidal stromal model can be integrated with RPE cells, choroidal melanocytes, and human umbilical vein endothelial cells to better recapitulate the environment of the native choroid. This model could help provide insight into RPE-choroid interactions and pathophysiological mechanisms affecting the choroid. Several hypotheses have been proposed regarding the relationship between RPE and choroid in AMD. One hypothesis for atrophic AMD is that RPE loss preceded the death of choriocapillaris (McLeod et al., 2009). RPE has been shown to produce VEGF, which is a vasodilator, endothelial cell survival factor, and angiogenic factor (Adamis et al., 1993). When VEGF is no longer in areas of RPE atrophy, choriocapillaris constricts or degenerates and eventually atrophies. However, choriocapillaris loss has also been observed in the absence of RPE atrophy in early AMD (Seddon A. W. R. et al., 2016), suggesting other factors are also involved. In contrast, the loss of choroidal vasculature may be the initial insult that causes RPE atrophy in neovascular AMD. Choriocapillaris degeneration results in hypoxia in RPE due to a reduced blood supply. It has been suggested that hypoxic RPE produces VEGF, which stimulates the formation of new vessels from choriocapillaris and results in choroidal neovascularization (Bhutto and Lutty, 2012; Seddon J. M. et al., 2016). Future studies using *in vitro* RPE-choroidal models could provide novel insights and help us determine the mechanism of tissue damage and disease progression in AMD.

Another method to engineer a layered retinal model is bioprinting, which has been increasingly used to create 3D tissue constructs. 3D bioprinter systems allow precise positioning and deposition of bio-components layer-by-layer, providing an attractive strategy to engineer complex tissues (Tasoglu and Demirci, 2013). A recent study by Masaeli et al. (2020) demonstrated the potential of using bioprinting to recreate retinal components. Using cells isolated from post-mortem donor eye, RPE cells were first cultured on a thin layer of gelatin methacrylate to mimic the Bruch’s membrane/RPE complex. Photoreceptors were then bioprinted onto RPE cell sheets, creating a layered structure. Notably, the authors showed that both RPE and photoreceptors retained expression of structural markers, while the RPE cells retain functional activities in VEGF secretion and phagocytosis (Masaeli et al., 2020). Future research that incorporates 3D bioprinting using retinal cells derived from stem cells would be important to overcome the problem with donor shortage to obtain cellular source for tissue engineering. Altogether, advances in pluripotent stem cells, biomaterials and 3D bioprinting provide new methods to develop better *in vitro* cell models that could accelerate functional study of AMD-associated genes and drug development.

## New Molecular Tools to Facilitate Functional Study of AMD-Associated Genes

Conventional strategies to study gene functions involve either gain-of-function studies by transgene overexpression, as well as loss-of-function studies by RNAi knockdown or homologous recombination-mediated knockout. The emergence of clustered regularly interspaced short palindromic repeats (CRISPR) technology provides exciting opportunities to facilitate functional studies of AMD-associated genes. Earlier use of CRISPR technology focuses on gene knockout for loss-of-function studies. In a widely used CRISPR system for gene editing, a guide RNA (gRNA) is used to direct the Cas9 nuclease derived from *Streptococcus pyogenes* (SpCas9) to cut a specific target DNA sequence and generate a double-strand DNA cleavage, which results in permanent and heritable gene knockout (Cho et al., 2013; Cong et al., 2013; Liu and Li, 2019). On the other hand, CRISPR/Cas can also be used for gene editing to correct SNPs at loci that confer high risks of AMD, such as the *CFH* locus using CRISPR-mediated base editing (Tran et al., 2019). Subsequent research have derived Cas9 nucleases from other organisms, such as SaCas9 derived from *Staphylococcus aureus* which is smaller in size and would facilitate packaging into viral vectors for gene delivery (Ran et al., 2015).

Beside gene editing, CRISPR can also be repurposed for gene activation or repression. In particular, a nuclease inactive or dead Cas9 (dCas9) can be fused with transcriptional activator(s) or repressor(s) to regulate gene expression, termed CRISPR activation (CRISPRa) or interference (CRISPRi), respectively (Boettcher and McManus, 2015). Numerous CRISPRa systems have been developed in recent years. The first generation of CRISPRa system consisted of dCas9 fused to a VP64 activator (Gilbert et al., 2013), which is capable of activating expression of silent genes or upregulating expression of active genes (Gilbert et al., 2013; Mali et al., 2013; Perez-Pinera et al., 2013). However, the activation levels observed with dCas9-VP64 in mammalian cells were relatively small using a single sgRNA. Subsequently, several improved CRISPRa systems have been developed, including VPR, SunTag, and SAM (Tanenbaum et al., 2014; Chavez et al., 2015, 2016; Konermann et al., 2015). The VPR system employs three potent activators VP64-p65-Rta fused to a dCas9. Similarly, the SAM system uses multiple transcriptional activators, dCas9-VP64 paired with MS2-p65-HSF1, to induce a synergistic effect. On the other hand, the SunTag system involves fusing dCas9 to a tandem array of peptides that can recruit multiple copies of the VP64 effector. Also, our group has reported a cloning-free pipeline that harnesses CRISPRa for gene activation, which can be used as a rapid workflow for gain-of-function studies (Fang et al., 2019). On the other hand, CRISPRi utilizes dCas9 that is fused to a repressor complex such as Krupper-associated box (KRAB), which allows repression of the target gene (Gilbert et al., 2013). An enhanced version of CRISPR repressor is dCas9-KRAB-MeCP2, which showed improved repression compared to dCas9-KRAB for the majority of genes tested (Yeo et al., 2018). Notably, CRISPRi systems are capable of multiplex gene repression, with high knockdown levels and minimal off-target effects (Housden and Perrimon, 2016). These technologies could be used to decipher functional roles of novel AMD-associated genes such as those identified in recent GWAS studies (Fritsche et al., 2016; Ratnapriya et al., 2019). Collectively, these CRISPRa and CRISPRi systems allow robust activation of multiple genes simultaneously with high efficiency and specificity, providing valuable tools for functional studies of AMD-associated genes.

Notably, CRISPRa and CRISPRi pooled libraries have been developed for genome-wide screens to study gene functions (Gilbert et al., 2014; Konermann et al., 2015). This provides an exciting strategy to identify novel genes that contribute to pathological processes leading to AMD, such as degeneration of RPE cells. In CRISPR pooled screens, the cells are transduced with a gRNA library to activate or inhibit a pool of target genes, followed by cell selection based on the phenotype of interest (e.g., RPE degeneration). Subsequently, the cell samples can be processed for next generation sequencing to identify the sgRNAs and the target genes that cause the phenotype of interest (Joung et al., 2017; Sanson et al., 2018). Genome-scale CRISPR functional screens offer a powerful modality to interrogate gene function that may take years to identify using conventional strategies. Future genome-wide screens using RPE models could enable identification of genes that are important in RPE degeneration and provide insights into AMD pathology.

Beyond gene function studies, CRISPRa and CRISPRi could also be applied to study regulatory regions of AMD genes. Since the majority of genetic variation associated with complex human disease is found within non-coding regions of the genome (Thurman et al., 2012), understanding how regulatory regions within the non-coding DNA regulate gene expression could help us understand pathogenesis of AMD. In this regard, CRISPRa or CRISPRi could be used to target non-coding regions of GWAS loci to identify novel regulatory regions of AMD-associated genes (Klann et al., 2018).

In addition to DNA-targeting CRISPR systems, RNA-targeting Cas9 enzymes are also available, such as CasRx which showed robust knockdown of gene expression (Konermann et al., 2018). Interestingly, CasRx can also be used to target pre-mRNA to manipulate alternative splicing (Konermann et al., 2018). Deregulation of alternative splicing has been implicated in the aging process (Li et al., 2017) and observed in several age-associated diseases such as amyotrophic lateral sclerosis and Alzheimer’s disease (Lin et al., 1998; Spillantini et al., 1998; Glatz et al., 2006). In regards to AMD, Allikmets et al. (1997) have shown that a point mutation (G5196A) in the Stargardt disease gene ABCA4, eliminates a 5′ donor splice site and increases the risk of AMD. However, a subsequent GWAS study with larger cohorts could not confirm this association between ABCA4 and AMD (Fritsche et al., 2016). Overall, the association of AMD pathophysiology with alternative splicing regulation remains unclear and CasRx technology could facilitate research in this understudied area. In summary, recent development of CRISPR/Cas technology has greatly expanded the toolbox to carry out functional study of AMD-associated genes, providing new tools that can modulate gene expression by targeting at the DNA level, RNA level as well as the splicing variants.

## Conclusion

Despite extensive research in AMD and considerable success in identifying genetic associations with this disease, we still do not fully understand the pathological mechanisms leading to AMD. Recent advances in the field of genomics, cell biology, molecular biology and tissue engineering offer an unprecedented opportunity for functional genomics studies and help to efficiently decipher the genetic contribution to AMD pathogenesis. Whilst identification of novel AMD-associated genes is greatly facilitated by new development in genome-wide analysis methods, more study is required to understand the functional role of these genes in AMD. Single cell transcriptomics offers a unique opportunity to understand how these genes affect specific cell types in the retina. Advances in stem cells and biomaterials provide better *in vitro* systems to study the function of AMD-related genes in the clinically relevant cell types, while new CRISPR technologies provide an efficient method to perform gain-of-function and loss-of-function studies for AMD-related genes. Future studies that utilize the technologies discussed here would provide novel insights into AMD genetics and accelerate identification of new therapeutic targets.

## Author Contributions

TN, CL, RG, and RCBW provided conceptual framework of this work. TN, DU, RHCL, CL, RG, and RCBW contributed to manuscript writing. All authors approved the manuscript.

## Conflict of Interest

The authors declare that the research was conducted in the absence of any commercial or financial relationships that could be construed as a potential conflict of interest.

## References

[B1] Ablonczy Z. Dahrouj M. Tang P. H. Liu Y. Sambamurti K. Marmorstein A. D. (2011). Human retinal pigment epithelium cells as functional models for the RPE in vivo. *Invest. Ophthalmol. Vis. Sci.* 52 8614–8620. 10.1167/iovs.11-8021 21960553 PMC3208409

[B2] Adamis A. P. Shima D. T. Yeo K. T. Yeo T. K. Brown L. F. Berse B. (1993). Synthesis and secretion of vascular permeability factor/vascular endothelial growth factor by human retinal pigment epithelial cells. *Biochem. Biophys. Res. Commun.* 193 631–638. 10.1006/bbrc.1993.1671 8512562

[B3] Allikmets R. Shroyer N. F. Singh N. Seddon J. M. Lewis R. A. Bernstein P. S. (1997). Mutation of the Stargardt disease gene (ABCR) in age-related macular degeneration. *Science* 277 1805–1807. 10.1126/science.277.5333.1805 9295268

[B4] Baldi A. De Luca A. Morini M. Battista T. Felsani A. Baldi F. (2002). The HtrA1 serine protease is down-regulated during human melanoma progression and represses growth of metastatic melanoma cells. *Oncogene* 21 6684–6688. 10.1038/sj.onc.1205911 12242667

[B5] Bhutto I. Lutty G. (2012). Understanding age-related macular degeneration (AMD): relationships between the photoreceptor/retinal pigment epithelium/Bruch’s membrane/choriocapillaris complex. *Mol. Aspects Med.* 33 295–317. 10.1016/j.mam.2012.04.005 22542780 PMC3392421

[B6] Blasiak J. Pawlowska E. Szczepanska J. Kaarniranta K. (2019). Interplay between autophagy and the ubiquitin-proteasome system and its role in the pathogenesis of age-related macular degeneration. *Int. J. Mol. Sci.* 20:210. 10.3390/ijms20010210 30626110 PMC6337628

[B7] Blythe E. E. Gates S. N. Deshaies R. J. Martin A. (2019). Multisystem proteinopathy mutations in VCP/p97 increase NPLOC4⋅UFD1L binding and substrate processing. *Structure* 27 1820.e4–1829.e4. 31623962 10.1016/j.str.2019.09.011 PMC6929323

[B8] Boettcher M. McManus M. T. (2015). Choosing the right tool for the job: RNAi, TALEN, or CRISPR. *Mol. Cell* 58 575–585. 10.1016/j.molcel.2015.04.028 26000843 PMC4441801

[B9] Brodie A. Azaria J. R. Ofran Y. (2016). How far from the SNP may the causative genes be? *Nucleic Acids Res.* 44 6046–6054. 10.1093/nar/gkw500 27269582 PMC5291268

[B10] Brown D. M. Michels M. Kaiser P. K. Heier J. S. Sy J. P. Ianchulev T. (2009). Ranibizumab versus verteporfin photodynamic therapy for neovascular age-related macular degeneration: two-year results of the ANCHOR study. *Ophthalmology* 116 57.e5–65.e5. 19118696 10.1016/j.ophtha.2008.10.018

[B11] Camelo S. (2014). Potential sources and roles of adaptive immunity in age-related macular degeneration: shall we rename AMD into autoimmune macular disease? *Autoimmune Dis.* 2014:532487. 10.1155/2014/532487 PMC4022009 24876950

[B12] Cannon M. E. Mohlke K. L. (2018). Deciphering the emerging complexities of molecular mechanisms at GWAS loci. *Am. J. Hum. Genet.* 103 637–653. 10.1016/j.ajhg.2018.10.001 30388398 PMC6218604

[B13] Chang M. He L. Cai L. (2018). An Overview of Genome-Wide Association Studies. *Methods Mol. Biol.* 1754 97–108. 29536439 10.1007/978-1-4939-7717-8_6

[B14] Chavez A. Scheiman J. Vora S. Pruitt B. W. Tuttle M. Iyer P. R. (2015). Highly efficient Cas9-mediated transcriptional programming. *Nat. Methods* 12 326–328. 10.1038/nmeth.3312 25730490 PMC4393883

[B15] Chavez A. Tuttle M. Pruitt B. W. Ewen-Campen B. Chari R. Ter-Ovanesyan D. (2016). Comparison of Cas9 activators in multiple species. *Nat. Methods* 13 563–567. 10.1038/nmeth.3871 27214048 PMC4927356

[B16] Chien J. Staub J. Hu S.-I. Erickson-Johnson M. R. Couch F. J. Smith D. I. (2004). A candidate tumor suppressor HtrA1 is downregulated in ovarian cancer. *Oncogene* 23 1636–1644. 10.1038/sj.onc.1207271 14716297

[B17] Chirco K. R. Sohn E. H. Stone E. M. Tucker B. A. Mullins R. F. (2017). Structural and molecular changes in the aging choroid: implications for age-related macular degeneration. *Eye* 31 10–25. 10.1038/eye.2016.216 27716746 PMC5233940

[B18] Cho S. W. Kim S. Kim J. M. Kim J.-S. (2013). Targeted genome engineering in human cells with the Cas9 RNA-guided endonuclease. *Nat. Biotechnol.* 31 230–232. 10.1038/nbt.2507 23360966

[B19] Clark S. J. Perveen R. Hakobyan S. Morgan B. P. Sim R. B. Bishop P. N. (2010). Impaired binding of the age-related macular degeneration-associated complement factor H 402H allotype to Bruch’s membrane in human retina. *J. Biol. Chem.* 285 30192–30202. 10.1074/jbc.m110.103986 20660596 PMC2943316

[B20] Colquitt J. L. Jones J. Tan S. C. Takeda A. Clegg A. J. Price A. (2008). Ranibizumab and pegaptanib for the treatment of age-related macular degeneration: a systematic review and economic evaluation. *Health Technol. Assess.* 12 3–4. 10.3310/hta12160 18462575

[B21] Cong L. Ran F. A. Cox D. Lin S. Barretto R. Habib N. (2013). Multiplex genome engineering using CRISPR/Cas systems. *Science* 339 819–823. 23287718 10.1126/science.1231143 PMC3795411

[B22] Cougnard-Grégoire A. Delyfer M.-N. Korobelnik J.-F. Rougier M.-B. Le Goff M. Dartigues J.-F. (2014). Elevated high-density lipoprotein cholesterol and age-related macular degeneration: the Alienor study. *PLoS One* 9:e90973. 10.1371/journal.pone.0090973 24608419 PMC3946623

[B23] Despriet D. D. G. Klaver C. C. W. Witteman J. C. M. Bergen A. A. B. Kardys I. de Maat M. P. M. (2006). Complement factor H polymorphism, complement activators, and risk of age-related macular degeneration. *JAMA* 296 301–309. 16849663 10.1001/jama.296.3.301

[B24] Djigo A. D. Bérubé J. Landreville S. Proulx S. (2019). Characterization of a tissue-engineered choroid. *Acta Biomater.* 84 305–316. 10.1016/j.actbio.2018.11.033 30476582

[B25] Edwards A. O. Ritter R. III Abel K. J. Manning A. Panhuysen C. Farrer L. A. (2005). Complement factor H polymorphism and age-related macular degeneration. *Science* 308 421–424. 10.1126/science.1110189 15761121

[B26] Eiraku M. Takata N. Ishibashi H. Kawada M. Sakakura E. Okuda S. (2011). Self-organizing optic-cup morphogenesis in three-dimensional culture. *Nature* 472 51–56. 10.1038/nature09941 21475194

[B27] Falcón-Pérez J. M. Starcevic M. Gautam R. Dell’Angelica E. C. (2002). BLOC-1, a novel complex containing the pallidin and muted proteins involved in the biogenesis of melanosomes and platelet-dense granules. *J. Biol. Chem.* 277 28191–28199. 10.1074/jbc.m204011200 12019270

[B28] Fang L. Hung S. S. C. Yek J. El Wazan L. Nguyen T. Khan S. (2019). A simple cloning-free method to efficiently induce gene expression using CRISPR/Cas9. *Mol. Ther. Nucleic Acids* 14 184–191. 10.1016/j.omtn.2018.11.008 30594894 PMC6307107

[B29] Farazdaghi M. K. Ebrahimi K. B. (2019). Role of the choroid in age-related macular degeneration: a current review. *J. Ophthalmic Vis. Res.* 14 78–87. 10.4103/jovr.jovr_125_18 30820291 PMC6388521

[B30] Farkas M. H. Grant G. R. White J. A. Sousa M. E. Consugar M. B. Pierce E. A. (2013). Transcriptome analyses of the human retina identify unprecedented transcript diversity and 3.5 Mb of novel transcribed sequence via significant alternative splicing and novel genes. *BMC Genomics* 14:486. 10.1186/1471-2164-14-486 23865674 PMC3924432

[B31] Finnemann S. C. Bonilha V. L. Marmorstein A. D. Rodriguez-Boulan E. (1997). Phagocytosis of rod outer segments by retinal pigment epithelial cells requires αvβ5 integrin for binding but not for internalization. *Proc. Natl. Acad. Sci. U.S.A.* 94 12932–12937. 10.1073/pnas.94.24.12932 9371778 PMC24241

[B32] Friedrich U. Myers C. A. Fritsche L. G. Milenkovich A. Wolf A. Corbo J. C. (2011). Risk- and non-risk-associated variants at the 10q26 AMD locus influence ARMS2 mRNA expression but exclude pathogenic effects due to protein deficiency. *Hum. Mol. Genet.* 20 1387–1399. 10.1093/hmg/ddr020 21252205 PMC3049360

[B33] Fritsche L. G. Chen W. Schu M. Yaspan B. L. Yu Y. Thorleifsson G. (2013). Seven new loci associated with age-related macular degeneration. *Nat. Genet.* 45 433–439. 10.1038/ng.2578 23455636 PMC3739472

[B34] Fritsche L. G. Igl W. Bailey J. N. C. Grassmann F. Sengupta S. Bragg-Gresham J. L. (2016). A large genome-wide association study of age-related macular degeneration highlights contributions of rare and common variants. *Nat. Genet.* 48 134–143. 26691988 10.1038/ng.3448 PMC4745342

[B35] Fritsche L. G. Loenhardt T. Janssen A. Fisher S. A. Rivera A. Keilhauer C. N. (2008). Age-related macular degeneration is associated with an unstable ARMS2 (LOC387715) mRNA. *Nat. Genet.* 40 892–896. 10.1038/ng.170 18511946

[B36] Galloway C. A. Dalvi S. Hung S. S. C. MacDonald L. A. Latchney L. R. Wong R. C. B. (2017). Drusen in patient-derived hiPSC-RPE models of macular dystrophies. *Proc. Natl. Acad. Sci. U.S.A.* 114 E8214–E8223. 28878022 10.1073/pnas.1710430114 PMC5625924

[B37] Gilbert L. A. Horlbeck M. A. Adamson B. Villalta J. E. Chen Y. Whitehead E. H. (2014). Genome-scale CRISPR-mediated control of gene repression and activation. *Cell* 159 647–661. 10.1016/j.cell.2014.09.029 25307932 PMC4253859

[B38] Gilbert L. A. Larson M. H. Morsut L. Liu Z. Brar G. A. Torres S. E. (2013). CRISPR-mediated modular RNA-guided regulation of transcription in eukaryotes. *Cell* 154 442–451. 10.1016/j.cell.2013.06.044 23849981 PMC3770145

[B39] Glatz D. C. Rujescu D. Tang Y. Berendt F. J. Hartmann A. M. Faltraco F. (2006). The alternative splicing of tau exon 10 and its regulatory proteins CLK2 and TRA2-BETA1 changes in sporadic Alzheimer’s disease. *J. Neurochem.* 96 635–644. 10.1111/j.1471-4159.2005.03552.x 16371011

[B40] Golestaneh N. Chu Y. Xiao Y.-Y. Stoleru G. L. Theos A. C. (2017). Dysfunctional autophagy in RPE, a contributing factor in age-related macular degeneration. *Cell Death Dis.* 8:e2537. 10.1038/cddis.2016.453 28055007 PMC5386365

[B41] Gonzalez-Cordero A. Kruczek K. Naeem A. Fernando M. Kloc M. Ribeiro J. (2017). Recapitulation of human retinal development from human pluripotent stem cells generates transplantable populations of cone photoreceptors. *Stem Cell Rep.* 9 820–837. 10.1016/j.stemcr.2017.07.022 28844659 PMC5599247

[B42] Graham J. R. Chamberland A. Lin Q. Li X. J. Dai D. Zeng W. (2013). Serine protease HTRA1 antagonizes transforming growth factor-β signaling by cleaving its receptors and loss of HTRA1 in vivo enhances bone formation. *PLoS One* 8:e74094. 10.1371/journal.pone.0074094 24040176 PMC3770692

[B43] Grassmann F. Heid I. M. Weber B. H. F. International Amd Genomics Consortium (Iamdgc). (2017). Recombinant haplotypes(narrow)the ARMS2/HTRA1 association signal for age-related macular degeneration. *Genetics* 205 919–924. 10.1534/genetics.116.195966 27879347 PMC5289859

[B44] Grau S. Baldi A. Bussani R. Tian X. Stefanescu R. Przybylski M. (2005). Implications of the serine protease HtrA1 in amyloid precursor protein processing. *Proc. Natl. Acad. Sci. U.S.A.* 102 6021–6026. 10.1073/pnas.0501823102 15855271 PMC1087941

[B45] Grau S. Richards P. J. Kerr B. Hughes C. Caterson B. Williams A. S. (2006). The role of human HtrA1 in arthritic disease. *J. Biol. Chem.* 281 6124–6129. 10.1074/jbc.m500361200 16377621

[B46] Grindberg R. V. Yee-Greenbaum J. L. McConnell M. J. Novotny M. O’Shaughnessy A. L. Lambert G. M. (2013). RNA-sequencing from single nuclei. *Proc. Natl. Acad. Sci. U.S.A.* 110 19802–19807. 24248345 10.1073/pnas.1319700110 PMC3856806

[B47] Gusev A. Ko A. Shi H. Bhatia G. Chung W. Penninx B. W. J. H. (2016). Integrative approaches for large-scale transcriptome-wide association studies. *Nat. Genet.* 48 245–252. 26854917 10.1038/ng.3506 PMC4767558

[B48] Guymer R. H. Brassington K. H. Dimitrov P. Makeyeva G. Plunkett M. Xia W. (2014). Nanosecond-laser application in intermediate AMD: 12-month results of fundus appearance and macular function. *Clin. Experiment. Ophthalmol.* 42 466–479. 10.1111/ceo.12247 24118741

[B49] Hadfield K. D. Rock C. F. Inkson C. A. Dallas S. L. Sudre L. Wallis G. A. (2008). HtrA1 inhibits mineral deposition by osteoblasts: requirement for the protease and PDZ domains. *J. Biol. Chem.* 283 5928–5938. 10.1074/jbc.m709299200 18156628

[B50] Hageman G. S. Anderson D. H. Johnson L. V. Hancox L. S. Taiber A. J. Hardisty L. I. (2005). A common haplotype in the complement regulatory gene factor H (HF1/CFH) predisposes individuals to age-related macular degeneration. *Proc. Natl. Acad. Sci. U.S.A.* 102 7227–7232. 10.1073/pnas.0501536102 15870199 PMC1088171

[B51] Haines J. L. Hauser M. A. Schmidt S. Scott W. K. Olson L. M. Gallins P. (2005). Complement factor H variant increases the risk of age-related macular degeneration. *Science* 308 419–421. 10.1126/science.1110359 15761120

[B52] Hallam D. Collin J. Bojic S. Chichagova V. Buskin A. Xu Y. (2017). An induced pluripotent stem cell patient specific model of complement factor H (Y402H) polymorphism displays characteristic features of age-related macular degeneration and indicates a beneficial role for UV light exposure. *Stem Cells* 35 2305–2320. 10.1002/stem.2708 28913923 PMC5698780

[B53] Han X. Gharahkhani P. Mitchell P. Liew G. Hewitt A. W. MacGregor S. (2020). Genome-wide meta-analysis identifies novel loci associated with age-related macular degeneration. *J. Hum. Genet.* 65 657–665. 10.1038/s10038-020-0750-x 32277175

[B54] Hartwig C. Monis W. J. Chen X. Dickman D. K. Pazour G. J. Faundez V. (2018). Neurodevelopmental disease mechanisms, primary cilia, and endosomes converge on the BLOC-1 and BORC complexes. *Dev. Neurobiol.* 78 311–330. 10.1002/dneu.22542 28986965 PMC5816705

[B55] Hasegawa Y. Takata N. Okuda S. Kawada M. Eiraku M. Sasai Y. (2016). Emergence of dorsal-ventral polarity in ESC-derived retinal tissue. *Development* 143 3895–3906. 10.1242/dev.134601 27633992

[B56] Hazim R. A. Volland S. Yen A. Burgess B. L. Williams D. S. (2019). Rapid differentiation of the human RPE cell line, ARPE-19, induced by nicotinamide. *Exp. Eye Res.* 179 18–24. 10.1016/j.exer.2018.10.009 30336127 PMC6360117

[B57] Hernández-Zimbrón L. F. Zamora-Alvarado R. Ochoa-De la Paz L. Velez-Montoya R. Zenteno E. Gulias-Cañizo R. (2018). Age-related macular degeneration: new paradigms for treatment and management of AMD. *Oxid. Med. Cell. Longev.* 2018:8374647. 10.1155/2018/8374647 PMC5816845 29484106

[B58] Hormozdiari F. van de Bunt M. Segrè A. V. Li X. Joo J. W. J. Bilow M. (2016). Colocalization of GWAS and eQTL signals detects target genes. *Am. J. Hum. Genet.* 99 1245–1260. 10.1016/j.ajhg.2016.10.003 27866706 PMC5142122

[B59] Hoshino A. Ratnapriya R. Brooks M. J. Chaitankar V. Wilken M. S. Zhang C. (2017). Molecular anatomy of the developing human retina. *Dev. Cell* 43 763.e4–779.e4. 10.1016/j.devcel.2017.10.029 29233477 PMC5776731

[B60] Housden B. E. Perrimon N. (2016). Comparing CRISPR and RNAi-based screening technologies. *Nat. Biotechnol.* 34 621–623. 10.1038/nbt.3599 27281421

[B61] Hung S. S. C. Khan S. Lo C. Y. Hewitt A. W. Wong R. C. B. (2017). Drug discovery using induced pluripotent stem cell models of neurodegenerative and ocular diseases. *Pharmacol. Ther.* 177 32–43. 10.1016/j.pharmthera.2017.02.026 28223228

[B62] Jaffe G. J. Ying G.-S. Toth C. A. Daniel E. Grunwald J. E. Martin D. F. (2019). Macular morphology and visual acuity in year five of the comparison of age-related macular degeneration treatments trials. *Ophthalmology* 126 252–260. 10.1016/j.ophtha.2018.08.035 30189282 PMC6462189

[B63] Jansen J. C. Timal S. van Scherpenzeel M. Michelakakis H. Vicogne D. Ashikov A. (2016). TMEM199 deficiency is a disorder of golgi homeostasis characterized by elevated aminotransferases, alkaline phosphatase, and cholesterol and abnormal glycosylation. *Am. J. Hum. Genet.* 98 322–330. 10.1016/j.ajhg.2015.12.011 26833330 PMC4746368

[B64] Jarrett S. G. Boulton M. E. (2012). Consequences of oxidative stress in age-related macular degeneration. *Mol. Aspects Med.* 33 399–417. 10.1016/j.mam.2012.03.009 22510306 PMC3392472

[B65] Joung J. Konermann S. Gootenberg J. S. Abudayyeh O. O. Platt R. J. Brigham M. D. (2017). Genome-scale CRISPR-Cas9 knockout and transcriptional activation screening. *Nat. Protoc.* 12 828–863. 28333914 10.1038/nprot.2017.016 PMC5526071

[B66] Kanda A. Chen W. Othman M. Branham K. E. H. Brooks M. Khanna R. (2007). A variant of mitochondrial protein LOC387715/ARMS2, not HTRA1, is strongly associated with age-related macular degeneration. *Proc. Natl. Acad. Sci. U.S.A.* 104 16227–16232. 10.1073/pnas.0703933104 17884985 PMC1987388

[B67] Kanda A. Stambolian D. Chen W. Curcio C. A. Abecasis G. R. Swaroop A. (2010). Age-related macular degeneration-associated variants at chromosome 10q26 do not significantly alter ARMS2 and HTRA1 transcript levels in the human retina. *Mol. Vis.* 16 1317–1323. 20664794 PMC2905635

[B68] Khan S. Hung S. S.-C. Wong R. C.-B. (2016). The use of induced pluripotent stem cells for studying and treating optic neuropathies. *Curr. Opin. Organ Transplant.* 21 484–489. 10.1097/mot.0000000000000348 27517502

[B69] Khanna S. Komati R. Eichenbaum D. A. Hariprasad I. Ciulla T. A. Hariprasad S. M. (2019). Current and upcoming anti-VEGF therapies and dosing strategies for the treatment of neovascular AMD: a comparative review. *BMJ Open Ophthalmol* 4:e000398. 10.1136/bmjophth-2019-000398 31909196 PMC6936465

[B70] Kiel C. Lastrucci C. Luthert P. J. Serrano L. (2017). Simple and complex retinal dystrophies are associated with profoundly different disease networks. *Sci. Rep.* 7:41835. 10.1038/srep41835 PMC5282568 28139756

[B71] Kim Y. Park H. Nah J. Moon S. Lee W. Hong S.-H. (2015). Essential role of POLDIP2 in Tau aggregation and neurotoxicity via autophagy/proteasome inhibition. *Biochem. Biophys. Res. Commun.* 462 112–118. 10.1016/j.bbrc.2015.04.084 25930997

[B72] Klann T. S. Black J. B. Gersbach C. A. (2018). CRISPR-based methods for high-throughput annotation of regulatory DNA. *Curr. Opin. Biotechnol.* 52 32–41. 10.1016/j.copbio.2018.02.004 29500989 PMC6082715

[B73] Klein B. E. Klein R. Linton K. L. (1992). Intraocular pressure in an American community. The Beaver Dam Eye Study. *Invest. Ophthalmol. Vis. Sci.* 33 2224–2228. 1607232

[B74] Klein R. J. Zeiss C. Chew E. Y. Tsai J.-Y. Sackler R. S. Haynes C. (2005). Complement factor H polymorphism in age-related macular degeneration. *Science* 308 385–389. 10.1126/science.1109557 15761122 PMC1512523

[B75] Konermann S. Brigham M. D. Trevino A. E. Joung J. Abudayyeh O. O. Barcena C. (2015). Genome-scale transcriptional activation by an engineered CRISPR-Cas9 complex. *Nature* 517 583–588. 10.1038/nature14136 25494202 PMC4420636

[B76] Konermann S. Lotfy P. Brideau N. J. Oki J. Shokhirev M. N. Hsu P. D. (2018). Transcriptome engineering with RNA-targeting type VI-D CRISPR effectors. *Cell* 173 665.e14–676.e14. 29551272 10.1016/j.cell.2018.02.033 PMC5910255

[B77] Kortvely E. Hauck S. M. Duetsch G. Gloeckner C. J. Kremmer E. Alge-Priglinger C. S. (2010). ARMS2 is a constituent of the extracellular matrix providing a link between familial and sporadic age-related macular degenerations. *Invest. Ophthalmol. Vis. Sci.* 51 79–88. 10.1167/iovs.09-3850 19696174

[B78] Kulkarni A. Anderson A. G. Merullo D. P. Konopka G. (2019). Beyond bulk: a review of single cell transcriptomics methodologies and applications. *Curr. Opin. Biotechnol.* 58 129–136. 30978643 10.1016/j.copbio.2019.03.001 PMC6710112

[B79] Kuznetsova A. V. Kurinov A. M. Aleksandrova M. A. (2014). Cell models to study regulation of cell transformation in pathologies of retinal pigment epithelium. *J. Ophthalmol.* 2014:801787. 10.1155/2014/801787 PMC4142280 25177495

[B80] Lake B. B. Codeluppi S. Yung Y. C. Gao D. Chun J. Kharchenko P. V. (2017). A comparative strategy for single-nucleus and single-cell transcriptomes confirms accuracy in predicted cell-type expression from nuclear RNA. *Sci. Rep.* 7:6031. 10.1038/s41598-017-04426-w PMC5519641 28729663

[B81] Langemeyer L. Ungermann C. (2015). BORC and BLOC-1: shared subunits in trafficking complexes. *Dev. Cell* 33 121–122. 10.1016/j.devcel.2015.04.008 25898163

[B82] Launay S. Maubert E. Lebeurrier N. Tennstaedt A. Campioni M. Docagne F. (2008). HtrA1-dependent proteolysis of TGF-β controls both neuronal maturation and developmental survival. *Cell Death Differ.* 15 1408–1416. 10.1038/cdd.2008.82 18551132

[B83] Li H. Wang Z. Ma T. Wei G. Ni T. (2017). Alternative splicing in aging and age-related diseases. *Transl. Med. Aging* 1 32–40. 10.1016/j.tma.2017.09.005

[B84] Liang Q. Dharmat R. Owen L. Shakoor A. Li Y. Kim S. (2019). Single-nuclei RNA-seq on human retinal tissue provides improved transcriptome profiling. *Nat. Commun.* 10:5743. 10.1038/s41467-019-12917-9 PMC6917696 31848347

[B85] Lidgerwood G. E. Lim S. Y. Crombie D. E. Ali R. Gill K. P. Hernández D. (2016). Defined medium conditions for the induction and expansion of human pluripotent stem cell-derived retinal pigment epithelium. *Stem Cell Rev. Rep.* 12 179–188. 10.1007/s12015-015-9636-2 26589197

[B86] Lin C. L. Bristol L. A. Jin L. Dykes-Hoberg M. Crawford T. Clawson L. (1998). Aberrant RNA processing in a neurodegenerative disease: the cause for absent EAAT2, a glutamate transporter, in amyotrophic lateral sclerosis. *Neuron* 20 589–602. 10.1016/s0896-6273(00)80997-6 9539131

[B87] Lin M. K. Yang J. Hsu C. W. Gore A. Bassuk A. G. Brown L. M. (2018). HTRA1, an age-related macular degeneration protease, processes extracellular matrix proteins EFEMP1 and TSP1. *Aging Cell* 17:e12710. 10.1111/acel.12710 29730901 PMC6052470

[B88] Liu J.-Q. Li T. (2019). CRISPR-Cas9-mediated loss-of-function screens. *Front. Life Sci.* 12 1–13. 10.1080/21553769.2019.1670739

[B89] Lukowski S. W. Lo C. Y. Sharov A. A. Nguyen Q. Fang L. Hung S. S. (2019). A single-cell transcriptome atlas of the adult human retina. *EMBO J.* 38:e100811. 10.15252/embj.2018100811 PMC6745503 31436334

[B90] Magnusson K. P. Duan S. Sigurdsson H. Petursson H. Yang Z. Zhao Y. (2006). CFH Y402H confers similar risk of soft drusen and both forms of advanced AMD. *PLoS Med.* 3:e5. 10.1371/journal.pmed.0030005 16300415 PMC1288033

[B91] Majewski J. Pastinen T. (2011). The study of eQTL variations by RNA-seq: from SNPs to phenotypes. *Trends Genet.* 27 72–79. 10.1016/j.tig.2010.10.006 21122937

[B92] Malek G. Yao P.-L. Choudhary M. (2020). “Models of pathologies associated with age-related macular degeneration and their utilities in drug discovery,” in *Drug Delivery Challenges and Novel Therapeutic Approaches for Retinal Diseases. Topics in Medicinal Chemistry*, ed. Cioffi C. L. (Cham: Springer), 1–41.

[B93] Mali P. Aach J. Stranges P. B. Esvelt K. M. Moosburner M. Kosuri S. (2013). CAS9 transcriptional activators for target specificity screening and paired nickases for cooperative genome engineering. *Nat. Biotechnol.* 31 833–838. 10.1038/nbt.2675 23907171 PMC3818127

[B94] Masaeli E. Forster V. Picaud S. Karamali F. Nasr-Esfahani M. H. Marquette C. (2020). Tissue engineering of retina through high resolution 3-dimensional inkjet bioprinting. *Biofabrication* 12:025006. 10.1088/1758-5090/ab4a20 31578006

[B95] Mauney J. Olsen B. R. Volloch V. (2010). Matrix remodeling as stem cell recruitment event: a novel in vitro model for homing of human bone marrow stromal cells to the site of injury shows crucial role of extracellular collagen matrix. *Matrix Biol.* 29 657–663. 10.1016/j.matbio.2010.08.008 20828613 PMC6817338

[B96] McCaughey T. Liang H. H. Chen C. Fenwick E. Rees G. Wong R. C. B. (2016). An interactive multimedia approach to improving informed consent for induced pluripotent stem cell research. *Cell Stem Cell* 18 307–308. 10.1016/j.stem.2016.02.006 26942850

[B97] McHugh K. J. Tao S. L. Saint-Geniez M. (2014). Porous poly(ε-caprolactone) scaffolds for retinal pigment epithelium transplantation. *Invest. Ophthalmol. Vis. Sci.* 55 1754–1762. 10.1167/iovs.13-12833 24550370 PMC3968933

[B98] McLeod D. S. Grebe R. Bhutto I. Merges C. Baba T. Lutty G. A. (2009). Relationship between RPE and choriocapillaris in age-related macular degeneration. *Invest. Ophthalmol. Vis. Sci.* 50 4982–4991. 10.1167/iovs.09-3639 19357355 PMC4829357

[B99] Menon M. Mohammadi S. Davila-Velderrain J. Goods B. A. Cadwell T. D. Xing Y. (2019). Single-cell transcriptomic atlas of the human retina identifies cell types associated with age-related macular degeneration. *Nat. Commun.* 10:4902. 10.1038/s41467-019-12780-8 PMC6814749 31653841

[B100] Micklisch S. Lin Y. Jacob S. Karlstetter M. Dannhausen K. Dasari P. (2017). Age-related macular degeneration associated polymorphism rs10490924 in ARMS2 results in deficiency of a complement activator. *J. Neuroinflammation* 14:4. 10.1186/s12974-016-0776-3 PMC5234120 28086806

[B101] Mijaljica D. Prescott M. Devenish R. J. (2011). V-ATPase engagement in autophagic processes. *Autophagy* 7 666–668. 21494095 10.4161/auto.7.6.15812

[B102] Miles A. L. Burr S. P. Grice G. L. Nathan J. A. (2017). The vacuolar-ATPase complex and assembly factors, TMEM199 and CCDC115, control HIF1α prolyl hydroxylation by regulating cellular iron levels. *eLife* 6:e22693. 10.7554/eLife.22693 28296633 PMC5391204

[B103] Mitchell P. Smith W. Attebo K. Wang J. J. (1995). Prevalence of age-related maculopathy in Australia. The blue mountains eye study. *Ophthalmology* 102 1450–1460. 9097791 10.1016/s0161-6420(95)30846-9

[B104] Mitter S. K. Rao H. V. Qi X. Cai J. Sugrue A. Dunn W. A. Jr. (2012). Autophagy in the retina: a potential role in age-related macular degeneration. *Adv. Exp. Med. Biol.* 723 83–90. 22183319 10.1007/978-1-4614-0631-0_12 PMC3638001

[B105] Mokhtarinia K. Nourbakhsh M. S. Masaeli E. Entezam M. Karamali F. Nasr-Esfahani M. H. (2018). Switchable phase transition behavior of thermoresponsive substrates for cell sheet engineering. *J. Polym. Sci. B Polym. Phys.* 56 1567–1576. 10.1002/polb.24744

[B106] Molins B. Fuentes-Prior P. Adán A. Antón R. Arostegui J. I. Yagüe J. (2016). Complement factor H binding of monomeric C-reactive protein downregulates proinflammatory activity and is impaired with at risk polymorphic CFH variants. *Sci. Rep.* 6:22889. 10.1038/srep22889 PMC4785391 26961257

[B107] Morohoshi K. Goodwin A. M. Ohbayashi M. Ono S. J. (2009). Autoimmunity in retinal degeneration: autoimmune retinopathy and age-related macular degeneration. *J. Autoimmun.* 33 247–254. 10.1016/j.jaut.2009.09.003 19846275

[B108] Mullany S. A. Moslemi-Kebria M. Rattan R. Khurana A. Clayton A. Ota T. (2011). Expression and functional significance of HtrA1 loss in endometrial cancer. *Clin. Cancer Res.* 17 427–436. 10.1158/1078-0432.ccr-09-3069 21098697 PMC3057564

[B109] Nakano T. Ando S. Takata N. Kawada M. Muguruma K. Sekiguchi K. (2012). Self-formation of optic cups and storable stratified neural retina from human ESCs. *Cell Stem Cell* 10 771–785. 10.1016/j.stem.2012.05.009 22704518

[B110] Ormsby R. J. Ranganathan S. Tong J. C. Griggs K. M. Dimasi D. P. Hewitt A. W. (2008). Functional and structural implications of the complement factor H Y402H polymorphism associated with age-related macular degeneration. *Invest. Ophthalmol. Vis. Sci.* 49 1763–1770. 10.1167/iovs.07-1297 18263814

[B111] Orozco L. D. Chen H.-H. Cox C. Katschke K. J. Jr. Arceo R. Espiritu C. (2020). Integration of eQTL and a single-cell atlas in the human eye identifies causal genes for age-related macular degeneration. *Cell Rep.* 30 1246.e6–1259.e6. 31995762 10.1016/j.celrep.2019.12.082

[B112] Paun C. C. Ersoy L. Schick T. Groenewoud J. M. M. Lechanteur Y. T. Fauser S. (2015). Genetic variants and systemic complement activation levels are associated with serum lipoprotein levels in age-related macular degeneration. *Invest. Ophthalmol. Vis. Sci.* 56 7766–7773. 10.1167/iovs.15-17035 26641553

[B113] Perez-Pinera P. Kocak D. D. Vockley C. M. Adler A. F. Kabadi A. M. Polstein L. R. (2013). RNA-guided gene activation by CRISPR-Cas9-based transcription factors. *Nat. Methods* 10 973–976. 10.1038/nmeth.2600 23892895 PMC3911785

[B114] Pinelli M. Carissimo A. Cutillo L. Lai C.-H. Mutarelli M. Moretti M. N. (2016). An atlas of gene expression and gene co-regulation in the human retina. *Nucleic Acids Res.* 44 5773–5784. 27235414 10.1093/nar/gkw486 PMC4937338

[B115] Ran F. A. Cong L. Yan W. X. Scott D. A. Gootenberg J. S. Kriz A. J. (2015). In vivo genome editing using *Staphylococcus aureus* Cas9. *Nature* 520 186–191. 25830891 10.1038/nature14299 PMC4393360

[B116] Ratnapriya R. Sosina O. A. Starostik M. R. Kwicklis M. Kapphahn R. J. Fritsche L. G. (2019). Retinal transcriptome and eQTL analyses identify genes associated with age-related macular degeneration. *Nat. Genet.* 51 606–610. 30742112 10.1038/s41588-019-0351-9 PMC6441365

[B117] Rivera A. Fisher S. A. Fritsche L. G. Keilhauer C. N. Lichtner P. Meitinger T. (2005). Hypothetical LOC387715 is a second major susceptibility gene for age-related macular degeneration, contributing independently of complement factor H to disease risk. *Hum. Mol. Genet.* 14 3227–3236. 16174643 10.1093/hmg/ddi353

[B118] Sanson K. R. Hanna R. E. Hegde M. Donovan K. F. Strand C. Sullender M. E. (2018). Optimized libraries for CRISPR-Cas9 genetic screens with multiple modalities. *Nat. Commun.* 9:5416. 10.1038/s41467-018-07901-8 PMC6303322 30575746

[B119] Seddon A. W. R. Macias-Fauria M. Long P. R. Benz D. Willis K. J. (2016). Sensitivity of global terrestrial ecosystems to climate variability. *Nature* 531 229–232. 26886790 10.1038/nature16986

[B120] Seddon J. M. McLeod D. S. Bhutto I. A. Villalonga M. B. Silver R. E. Wenick A. S. (2016). Histopathological insights into choroidal vascular loss in clinically documented cases of age-related macular degeneration. *JAMA Ophthalmol.* 134 1272–1280. 27657855 10.1001/jamaophthalmol.2016.3519 PMC6014730

[B121] Shaw P. X. Zhang L. Zhang M. Du H. Zhao L. Lee C. (2012). Complement factor H genotypes impact risk of age-related macular degeneration by interaction with oxidized phospholipids. *Proc. Natl. Acad. Sci. U.S.A.* 109 13757–13762. 22875704 10.1073/pnas.1121309109 PMC3427125

[B122] Shokoohmand A. Jeon J. E. Theodoropoulos C. Baldwin J. G. Hutmacher D. W. Feigl B. (2017). A novel 3D cultured model for studying early changes in age-related macular degeneration. *Macromol. Biosci.* 17:201700221. 10.1002/mabi.201700221 29076662

[B123] Skerka C. Lauer N. Weinberger A. A. W. A. Keilhauer C. N. Sühnel J. Smith R. (2007). Defective complement control of factor H (Y402H) and FHL-1 in age-related macular degeneration. *Mol. Immunol.* 44 3398–3406. 17399790 10.1016/j.molimm.2007.02.012

[B124] Smith E. N. D’Antonio-Chronowska A. Greenwald W. W. Borja V. Aguiar L. R. Pogue R. (2019). Human iPSC-derived retinal pigment epithelium: a model system for prioritizing and functionally characterizing causal variants at AMD risk loci. *Stem Cell Rep.* 12 1342–1353. 10.1016/j.stemcr.2019.04.012 PMC6565613 31080113

[B125] Souied E. H. Leveziel N. Richard F. Dragon-Durey M.-A. Coscas G. Soubrane G. (2005). Y402H complement factor H polymorphism associated with exudative age-related macular degeneration in the French population. *Mol. Vis.* 11 1135–1140. 16379025

[B126] Spillantini M. G. Murrell J. R. Goedert M. Farlow M. R. Klug A. Ghetti B. (1998). Mutation in the tau gene in familial multiple system tauopathy with presenile dementia. *Proc. Natl. Acad. Sci. U.S.A.* 95 7737–7741. 9636220 10.1073/pnas.95.13.7737 PMC22742

[B127] Strunz T. Lauwen S. Kiel C. International Amd Genomics Consortium (Iamdgc), Hollander A. Weber B. H. (2020). A transcriptome-wide association study based on 27 tissues identifies 106 genes potentially relevant for disease pathology in age-related macular degeneration. *Sci. Rep.* 10:1584. 10.1038/s41598-020-58510-9 PMC6994629 32005911

[B128] Supanji, Shimomachi M. Hasan M. Z. Kawaichi M. Oka C. (2013). HtrA1 is induced by oxidative stress and enhances cell senescence through p38 MAPK pathway. *Exp. Eye Res.* 112 79–92. 23623979 10.1016/j.exer.2013.04.013

[B129] Tam V. Patel N. Turcotte M. Bossé Y. Paré G. Meyre D. (2019). Benefits and limitations of genome-wide association studies. *Nat. Rev. Genet.* 20 467–484. 31068683 10.1038/s41576-019-0127-1

[B130] Tanenbaum M. E. Gilbert L. A. Qi L. S. Weissman J. S. Vale R. D. (2014). A protein-tagging system for signal amplification in gene expression and fluorescence imaging. *Cell* 159 635–646. 25307933 10.1016/j.cell.2014.09.039 PMC4252608

[B131] Tasoglu S. Demirci U. (2013). Bioprinting for stem cell research. *Trends Biotechnol.* 31 10–19. 23260439 10.1016/j.tibtech.2012.10.005 PMC3534918

[B132] Thurman R. E. Rynes E. Humbert R. Vierstra J. Maurano M. T. Haugen E. (2012). The accessible chromatin landscape of the human genome. *Nature* 489 75–82. 22955617 10.1038/nature11232 PMC3721348

[B133] Tosi G. M. Caldi E. Neri G. Nuti E. Marigliani D. Baiocchi S. (2017). HTRA1 and TGF-β1 concentrations in the aqueous humor of patients with neovascular age-related macular degeneration. *Invest. Ophthalmol. Vis. Sci.* 58 162–167. 28114575 10.1167/iovs.16-20922

[B134] Tran M. T. N. Khalid M. K. N. M. Pébay A. Cook A. L. Liang H. H. Wong R. C. B. (2019). Screening of CRISPR/Cas base editors to target the AMD high-risk Y402H complement factor H variant. *Mol. Vis.* 25 174–182. 30996586 PMC6441356

[B135] Trese M. Regatieri C. V. Young M. J. (2012). Advances in retinal tissue engineering. *Materials* 5 108–120. 28817034 10.3390/ma5010108 PMC5448948

[B136] Velez-Montoya R. Oliver S. C. N. Olson J. L. Fine S. L. Quiroz-Mercado H. Mandava N. (2014). Current knowledge and trends in age-related macular degeneration: genetics, epidemiology, and prevention. *Retina* 34 423–441. 24285245 10.1097/IAE.0000000000000036

[B137] Vierkotten S. Muether P. S. Fauser S. (2011). Overexpression of HTRA1 leads to ultrastructural changes in the elastic layer of Bruch’s membrane via cleavage of extracellular matrix components. *PLoS One* 6:e22959. 10.1371/journal.pone.0022959 21829675 PMC3149070

[B138] Voigt A. P. Whitmore S. S. Flamme-Wiese M. J. Riker M. J. Wiley L. A. Tucker B. A. (2019). Molecular characterization of foveal versus peripheral human retina by single-cell RNA sequencing. *Exp. Eye Res.* 184 234–242. 31075224 10.1016/j.exer.2019.05.001 PMC6596422

[B139] Völkner M. Zschätzsch M. Rostovskaya M. Overall R. W. Busskamp V. Anastassiadis K. (2016). Retinal Organoids from Pluripotent Stem Cells Efficiently Recapitulate Retinogenesis. *Stem Cell Rep.* 6 525–538. 10.1016/j.stemcr.2016.03.001 PMC4834051 27050948

[B140] Wainberg M. Sinnott-Armstrong N. Mancuso N. Barbeira A. N. Knowles D. A. Golan D. (2019). Opportunities and challenges for transcriptome-wide association studies. *Nat. Genet.* 51 592–599. 30926968 10.1038/s41588-019-0385-z PMC6777347

[B141] Weismann D. Hartvigsen K. Lauer N. Bennett K. L. Scholl H. P. N. Charbel Issa P. (2011). Complement factor H binds malondialdehyde epitopes and protects from oxidative stress. *Nature* 478 76–81. 21979047 10.1038/nature10449 PMC4826616

[B142] Whitmore S. S. Wagner A. H. DeLuca A. P. Drack A. V. Stone E. M. Tucker B. A. (2014). Transcriptomic analysis across nasal, temporal, and macular regions of human neural retina and RPE/choroid by RNA-Seq. *Exp. Eye Res.* 129 93–106. 25446321 10.1016/j.exer.2014.11.001 PMC4259842

[B143] Wong R. C. B. Lim S. Y. Hung S. S. C. Jackson S. Khan S. Van Bergen N. J. (2017). Mitochondrial replacement in an iPSC model of Leber’s hereditary optic neuropathy. *Aging* 9 1341–1350. 28455970 10.18632/aging.101231 PMC5425131

[B144] Wong W. L. Su X. Li X. Cheung C. M. G. Klein R. Cheng C.-Y. (2014). Global prevalence of age-related macular degeneration and disease burden projection for 2020 and 2040: a systematic review and meta-analysis. *Lancet Glob. Health* 2 e106–e116. 25104651 10.1016/S2214-109X(13)70145-1

[B145] Xiang P. Wu K.-C. Zhu Y. Xiang L. Li C. Chen D.-L. (2014). A novel Bruch’s membrane-mimetic electrospun substrate scaffold for human retinal pigment epithelium cells. *Biomaterials* 35 9777–9788. 25220295 10.1016/j.biomaterials.2014.08.040

[B146] Xu Y.-T. Wang Y. Chen P. Xu H.-F. (2012). Age-related maculopathy susceptibility 2 participates in the phagocytosis functions of the retinal pigment epithelium. *Int. J. Ophthalmol.* 5 125–132. 22762035 10.3980/j.issn.2222-3959.2012.02.02 PMC3359023

[B147] Yang Z. Tong Z. Chen Y. Zeng J. Lu F. Sun X. (2010). Genetic and functional dissection of HTRA1 and LOC387715 in age-related macular degeneration. *PLoS Genet.* 6:e1000836. 10.1371/journal.pgen.1000836 20140183 PMC2816682

[B148] Yeo N. C. Chavez A. Lance-Byrne A. Chan Y. Menn D. Milanova D. (2018). An enhanced CRISPR repressor for targeted mammalian gene regulation. *Nat. Methods* 15 611–616. 30013045 10.1038/s41592-018-0048-5 PMC6129399

[B149] Yu J. Wiita P. Kawaguchi R. Honda J. Jorgensen A. Zhang K. (2007). Biochemical analysis of a common human polymorphism associated with age-related macular degeneration. *Biochemistry* 46 8451–8461. 17580967 10.1021/bi700459a

[B150] Zareparsi S. Branham K. E. H. Li M. Shah S. Klein R. J. Ott J. (2005). Strong association of the Y402H variant in complement factor H at 1q32 with susceptibility to age-related macular degeneration. *Am. J. Hum. Genet.* 77 149–153. 15895326 10.1086/431426 PMC1226187

[B151] Zhong X. Gutierrez C. Xue T. Hampton C. Vergara M. N. Cao L.-H. (2014). Generation of three-dimensional retinal tissue with functional photoreceptors from human iPSCs. *Nat. Commun.* 5:4047. 10.1038/ncomms5047 PMC4370190 24915161

[B152] Zhou S. Flamier A. Abdouh M. Tétreault N. Barabino A. Wadhwa S. (2015). Differentiation of human embryonic stem cells into cone photoreceptors through simultaneous inhibition of BMP, TGFβ and Wnt signaling. *Development* 142 3294–3306. 26443633 10.1242/dev.125385

[B153] Zhu Z. Zhang F. Hu H. Bakshi A. Robinson M. R. Powell J. E. (2016). Integration of summary data from GWAS and eQTL studies predicts complex trait gene targets. *Nat. Genet.* 48 481–487. 27019110 10.1038/ng.3538

